# LONGITUDINAL ASSOCIATIONS BETWEEN PERSONAL GROWTH AND COGNITIVE FUNCTIONING IN ADULTHOOD

**DOI:** 10.1093/geroni/igac059.2078

**Published:** 2022-12-20

**Authors:** Masahiro Toyama

**Affiliations:** Marshall University, Barboursville, West Virginia, United States

## Abstract

While personal growth has been found to be associated with multiple aspects of health in adulthood, its associations with cognitive functioning have not been fully understood. The present study aimed to assess both directions of such longitudinal associations. Using data from the second wave (T1) and third wave (T2) of the Midlife in the United States (MIDUS) study (N = 4,206; mean age = 56.0 [SD = 12.3]), a longitudinal measurement model containing latent variables of episodic memory and executive function was first constructed. Built on the measurement model, a structural equation model was analyzed to assess cross-lagged relationships between personal growth and the two areas of cognitive functioning, in which T1 personal growth predicted residualized changes in episodic memory and executive function, and T1 episodic memory and executive function predicted change in personal growth, controlling for covariates. The results indicated that T1 personal growth significantly predicted smaller decreases in episodic memory, whereas it did not predict change in executive function. T1 episodic memory, but not T1 executive function, significantly predicted smaller decreases in personal growth. The present findings were unique, particularly implying potential longitudinal reciprocity between personal growth and episodic memory. These findings and implications can inform future research aimed at exploring approaches to promoting personal growth and cognitive functioning among aging adults.

